# Uptake of Home-Based Voluntary HIV Testing in Sub-Saharan Africa: A Systematic Review and Meta-Analysis

**DOI:** 10.1371/journal.pmed.1001351

**Published:** 2012-12-04

**Authors:** Kalpana Sabapathy, Rafael Van den Bergh, Sarah Fidler, Richard Hayes, Nathan Ford

**Affiliations:** 1 London School of Hygiene and Tropical Medicine, London, United Kingdom; 2 Médecins Sans Frontières, Brussels, Belgium; 3 Imperial College London, London, United Kingdom; 4 Médecins Sans Frontières, Geneva, Switzerland; 5 University of Cape Town, Cape Town, South Africa; Centers for Disease Control and Prevention, United States of America

## Abstract

**Introduction:**

Improving access to HIV testing is a key priority in scaling up HIV treatment and prevention services. Home-based voluntary counselling and testing (HBT) as an approach to delivering wide-scale HIV testing is explored here.

**Methods and Findings:**

We conducted a systematic review and random-effects meta-analysis of studies published between 1 January 2000 and 24 September 2012 that reported on uptake of HBT in sub-Saharan Africa, to assess the proportion of individuals accepting HBT and receiving their test result.

Our initial search yielded 1,199 articles; 114 were reviewed as full-text articles, and 19 publications involving 21 studies (*n* = 524,867 individuals offered HBT) were included for final review and meta-analysis. The studies came from five countries: Uganda, Malawi, Kenya, South Africa, and Zambia.

The proportion of people who accepted HBT (*n* = 474,377) ranged from 58.1% to 99.8%, with a pooled proportion of 83.3% (95% CI: 80.4%–86.1%). Heterogeneity was high (τ^2^ = 0.11). Sixteen studies reported on the number of people who received the result of HBT (*n* = 432,835). The proportion of individuals receiving their results out of all those offered testing ranged from 24.9% to 99.7%, with a pooled proportion of 76.7% (95% CI: 73.4%–80.0%) (τ^2^ = 0.12). HIV prevalence ranged from 2.9% to 36.5%. New diagnosis of HIV following HBT ranged from 40% to 79% of those testing positive. Forty-eight percent of the individuals offered testing were men, and they were just as likely to accept HBT as women (pooled odds ratio = 0.84; 95% CI: 0.56–1.26) (τ^2^ = 0.33). The proportion of individuals previously tested for HIV among those offered a test ranged from 5% to 66%. Studies in which <30% of individuals had been previously tested, local HIV prevalence was <10%, incentives were provided, or HBT was offered to household members of HIV-positive individuals showed higher uptake of testing. No evidence was reported of negative consequences of HBT.

**Conclusions:**

HBT could substantially increase awareness of HIV status in previously undiagnosed individuals in sub-Saharan Africa, with over three-quarters of the studies in this review reporting >70% uptake. It could be a valuable tool for treatment and prevention efforts.

*Please see later in the article for the Editors' Summary*

## Abstract 2

Kalpana Sabapathy and colleagues conduct a systematic review and meta-analysis to assess the acceptability of home-based voluntary counseling and testing for HIV in sub-Saharan Africa with some encouraging results.

## Editors' Summary

**Background:**

Knowledge of HIV status is crucial for both the prevention and treatment of HIV. However, according to the Joint United Nations Programme on HIV/AIDS (the UN agency responsible for HIV/AIDS), in low-and-middle-income countries only ten percent of those who need voluntary counseling and testing, because they may have been exposed to HIV infection, have access to this service. Even in health care settings in which voluntary counseling and HIV testing is routinely offered, such as to pregnant women, the number of people who use these services is low. This situation is partly because of the stigma and discrimination associated with HIV, which makes people reluctant to volunteer to come forward to be tested for HIV. To help overcome this problem, one important strategy in encouraging people to be tested for HIV is to offer them the opportunity to be counseled and tested at home—home-based voluntary counseling and testing (HBT). Using the HBT approach, people are visited in their home by health workers regardless of their perceived risk of HIV. HBT has obvious advantages and upholds the “3 Cs” principles of HIV testing: that testing is confidential, accompanied by counseling, and conducted only with informed consent.

**Why Was This Study Done?:**

The HBT approach has received widespread international support, and the World Health Organization has recently published guidance to service providers and policy makers about the delivery of HBT. However, the acceptability of HBT, that is, whether those offered HBT actually take up the offer and are tested, remains unknown, especially in sub-Saharan Africa, the world region with the highest prevalence of HIV. So, in this study, the researchers systematically compiled all of the available studies on this topic from sub-Saharan Africa to determine the acceptability of HBT and also to and identify any factors associated with the uptake of HBT.

**What Did the Researchers Do and Find?:**

The researchers searched several databases to identify suitable peer-reviewed studies from Africa published between January 2000 and September 2012. The researchers included studies that described any intervention to provide HIV testing at home and also reported the proportions of participants accepting HIV testing out of all individuals offered a home-based HIV test. Because different types of studies were included (such as randomized controlled trials, observational cohort studies, and cross-sectional surveys), the researchers tested the quality of included studies. Then they pooled all of the studies together to calculate the overall proportion of people who accepted HIV testing at home and the proportion who received their result.

Using these methods, the researchers included 21 studies from five African countries: Kenya, Malawi, South Africa, Uganda, and Zambia, comprising a total of 524,867 people. Overall, the proportion of people who accepted HBT ranged from 58.1% to 99.7%, with a pooled proportion of 83.3% accepting HBT (474,377 people). In the eight studies that separated data by gender, men were as likely as women to accept testing (78.5% versus 81.5%). Over three-quarters of everyone who accepted HBT received their result (77% in 16 studies reporting on this), and, importantly, the proportion of people with previously undiagnosed HIV was high (40%–79% of those diagnosed HIV-positive), emphasizing the value of HBT. The researchers also found that providing incentives, local HIV prevalence being less than 10%, and targeting HBT to household members of HIV-positive individuals may be factors associated with increased uptake of HBT, but further research is needed to verify the results of this subgroup analysis.

**What Do These Findings Mean?:**

These findings suggest that voluntary counseling and testing for HIV at home is highly acceptable in five countries in sub-Saharan Africa, with the majority of those tested receiving their test result, highlighting the importance of this approach in the diagnosis of HIV. Therefore, by increasing uptake of testing, HBT may provide an effective tool for governments and health service providers to increase access to HIV treatment and prevention. However, testing is just the first step in the management of HIV, and this study does not address the follow-up of those who tested positive using the home-based approach, such as access to treatment, as well as repeated HBT for ongoing knowledge of HIV status. The option of self-testing was examined in only one of the studies included in this review, but the researchers identify that self-testing at home with the support HBT staff is an important area of future research. Overall, HBT has the potential to substantially increase awareness of HIV status in previously undiagnosed men and women in sub-Saharan Africa.

**Additional Information:**

Please access these websites via the online version of this summary at http://dx.doi.org/10.1371/journal.pmed.1001351.

- The World Health Organization provides extensive information on HIV testing and counseling, and the World Health Organization's guidance on home-based testing mentioned in this summary is also available
- The Joint United Nations Programme on HIV/AIDS gives the latest facts and figures about the global status of HIV and about reducing stigma and discrimination around HIV

## Introduction

Testing for HIV is the first step in the cascade of care for HIV-positive individuals who need treatment. Knowledge of HIV status is also an important part of HIV prevention, for both HIV-negative and HIV-positive individuals, and developing innovative means to increase uptake of testing has recently been identified as an international policy priority [1]–[4]. Despite some progress, knowledge of HIV status remains low in sub-Saharan Africa (SSA), where HIV prevalence is highest [5]. National population surveys in six sub-Saharan African countries found that amongst participants living with HIV there was a wide range in the proportion of respondents aware of their status (from 31% in Congo to 69% in Kenya) [5]. Men have consistently been shown to be less likely to have been tested for HIV than women [5],[6].

Out-of-facility approaches to offering testing in the community [7],[8] and the workplace [9],[10] are means of bringing access to testing closer to clients. By removing distance as a barrier, these methods could be complementary means to scale up HIV testing [11]. Home-based voluntary counselling and testing (HBT) has been suggested as an effective out-of-facility approach for identifying HIV-infected people at an earlier stage of their disease and for enrolling them into care and treatment in a timely manner [12]–[14]. The World Health Organization has recently published a handbook to guide service providers and policy makers in delivering HBT [14]. HBT may reach individuals that community venue-based testing and workplace testing do not because it does not require clients to come forward [14]. In HBT it is the test provider who approaches the client, regardless of his/her perceived risk of having HIV [15]. However, there is uncertainty about HBT and concern that it may be poorly accepted or even harmful, partly owing to the enduring climate of stigma and discrimination around HIV/AIDS in many settings [16],[17].

We carried out a systematic review and meta-analysis of the available evidence regarding the acceptability of HBT in SSA, and assessed a number of potential determinants of uptake and programme success.

## Methods

We conducted this systematic review and meta-analysis based on a pre-defined search protocol (Text S2) that conformed to the criteria set out by the Meta-Analysis of Observational Studies in Epidemiology (MOOSE) group [18] and was in accordance with the PRISMA statement (Text S1). The specific objectives of the study were to summarise the following proportions: “accepted” (or uptake), defined as the proportion of all individuals offered HBT who accepted and had an HIV test performed at home; “received”, defined as the proportion of all individuals who accepted a home-based HIV test who subsequently obtained the result of the test; and “overall”, defined as the proportion of individuals who received a test result among all those offered HBT (including refusals). We also planned sub-group analyses as outlined below.

### Search Strategy

We aimed to summarise studies that described uptake of HIV testing provided at home in SSA. We screened studies published between 1 January 2000 (the onset of programmes providing antiretroviral therapy in SSA) and 24 September 2012. The following study designs were permitted: randomised controlled trials, observational cohort studies, cross-sectional surveys, and programme evaluations. Any study that described an intervention to provide HIV testing at home and reported proportions accepting HIV testing out of all individuals offered a home-based HIV test was included. Where acceptance of testing was reported, it was assumed that testing was performed unless stated otherwise.

To avoid duplication we excluded reports that pooled data from previously published studies, and where there was substantial overlap of study participants, we included the study with the most complete information. No language or age restriction was applied to the literature search. To identify studies for this systematic review, we searched the online databases PubMed, Embase, and Global Health (Ovid) and manually searched the bibliographies of relevant articles. We included only peer-reviewed journal articles; conference abstracts were excluded. Anticipating overlap between studies reporting HBT and other community-based strategies, we developed a broad compound search strategy that combined terms for “HIV”, “voluntary counselling and testing”, “home based”, “mobile”, “community”, “workplace”, “couples”, and “self”. We then combined these terms with the individual names of countries in SSA (Text S2). Finally, we excluded all studies that did not report home-based delivery of HIV testing.

Eligibility of abstracts and journal articles was determined by one investigator (K. S.) and verified by a second researcher (R. V. d. B.). Two investigators (K. S. and R. V. d. B.) then independently extracted data on study characteristics and outcomes using a standardised form. Any disagreements regarding eligibility or outcome data were resolved by a third investigator (N. F.). The rigour of study processes and research methods was examined using pre-defined criteria, but studies were not excluded for quality reasons.

### Data Synthesis and Analysis

We calculated the proportion of people who accepted HIV testing at home, and the proportion who received their test result out of those (i) who were offered and (ii) who accepted testing. The variance of raw proportions was stabilised using a Freeman–Tukey arcsine square-root transformation, and proportions were then pooled using a DerSimonian–Laird random-effects model [19]–[21]. Pooled odds ratios were calculated for proportions stratified by gender, also using a random-effects model. We report the *I* ^2^ statistic to assess the proportion of variability due to between-study heterogeneity, but as this estimate is known to increase as the number of participants contributing to the meta-analysis increases [21], we also report τ^2^ as a measure of between-study variance (reported on the arcsine square-root scale). We explored potential sources of heterogeneity through univariate sub-group analysis to determine the potential influence of the following covariates: HIV prevalence (<10% versus ≥10%), study period (before 2005 versus 2005 or later), incentives provided (yes versus no), sensitisation campaigns done (yes versus no), and study setting (urban versus rural). We further explored the potential influence of type of test (point-of-care testing with immediate result versus testing without immediate provision of result). Finally, sub-group analyses were done to assess the potential influence of the proportion of individuals in the studies who had been previously tested (arbitrarily divided into two categories, <30% versus ≥30%), and of studies that targeted HBT to household members of index HIV-positive individuals. We opted for sub-group analyses over meta-regression because of the limited number of studies and the dichotomous nature of most variables. All analyses were conducted using Stata version 12.0 (Stata Corp).

## Results

### Characteristics of Included Studies

**Figure 1:**
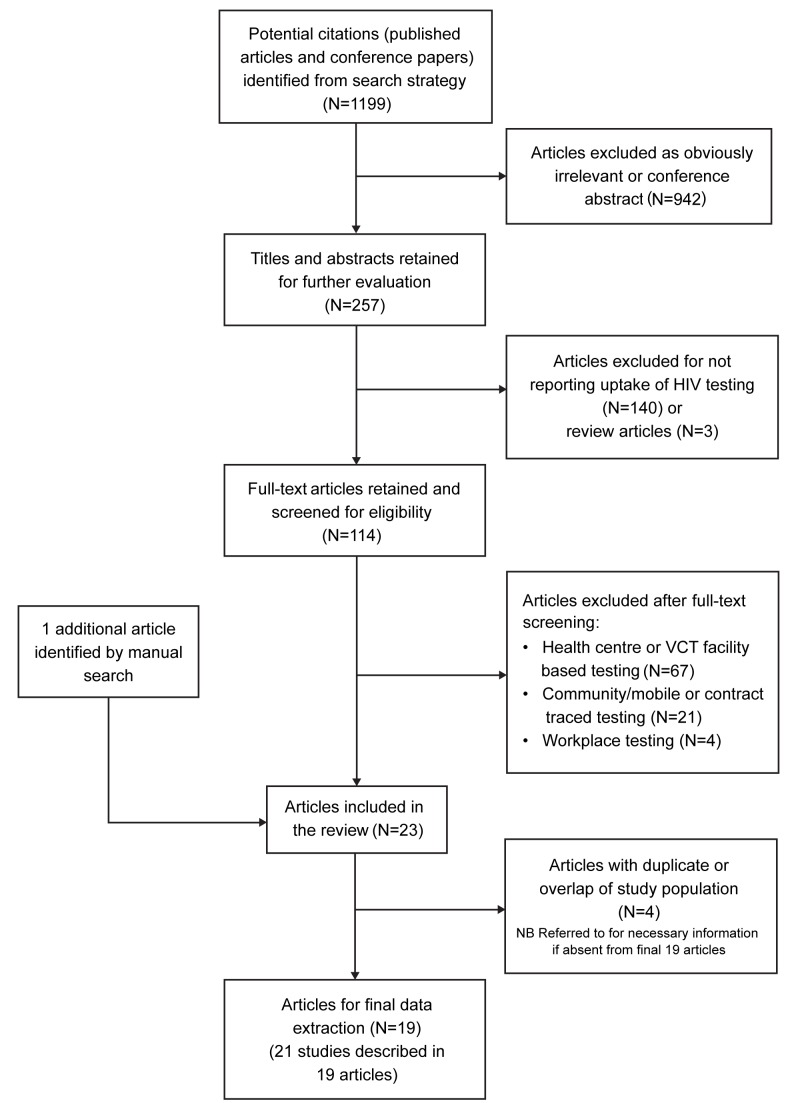
Flow diagram of study selection process.

Our initial search yielded 1,199 articles, of which 114 were reviewed as full-text articles and 19 were included in the meta-analysis (Figure 1) after excluding four studies with clearly overlapping study populations [22]–[25]. Two included publications presented data of two sub-studies: the first article included data from two surveys done in two separate time periods [26]; the second article reported different subsets of individuals (residents and migrants) [27]. As such, we present data and results of analyses based on these 21 studies from the 19 articles. The studies were from five countries: Uganda [28]–[35], Malawi [26],[36]–[39], Kenya [40],[41], South Africa [27],[42], and Zambia [43], and were carried out between 1999 and 2010. Most studies focused on adults (defined either as aged ≥18 y or, more commonly, ≥15 y), while seven studies also included children [28],[30],[32],[33],[35],[40],[42]. Regional HIV prevalence (reported by the authors for the study areas or obtained from Joint United Nations Programme on HIV/AIDS contemporaneous national data) ranged from 4.4% to 22% (Table 1). Testing was generally provided by counsellors; one study included laboratory assistants in the testing teams [32], and two utilised nurses [31],[42]. One study employed self-testing with counsellor supervision [36]. HIV prevalence amongst those tested ranged from 2.9% to 36.5%.

**Table 1:** Characteristics of included studies.

First Author, Publication Year	Country, Setting	Period of Study	Number Offered Testing	Purpose of Study	HIV Prevalence^a^	Age Eligibility of Participants	Testing Provider	Community Sensitisation Described	Incentives Provided	Sampling Method and Tests Used	Percent Previously Tested
Angotti (1), 2009	Malawi, three rural districts	2004	3,659	Longitudinal HIV prevalence study	4.4%–7.9%	15–49 y	Locally trained VCT counsellors	Yes	No	Oral swab (Orasure) (2004)	Not specified
Angotti (2), 2009	As above	2006	3,459	As above	As above	As above	As above	As above	As above	FP RDTs (Determine and UniGold) (2006)	66%
Choko, 2011	Malawi, urban district	2010	216	Feasibility of (supervised) oral self-testing	*11%*	22–32 y	Self-administered (supervision from VCT counsellor)	No	No	Oral swab (Oraquick) followed by FP RDTs (Determine and UniGold)	63%
Helleringer, 2009	Malawi, rural district	2006	751	Uptake of HBT	*11%*	18–35 y	Trained health counsellors	Yes	Yes—bar of soap	FP RDTs (Determine and UniGold)	21%
Kimaiyo, 2010	Kenya, two rural districts	2007–2009	101,167	Feasibility and acceptability of HBT	*6.3%*	>13 y and eligible children^b^	Counsellors trained for purpose	Yes	No	FP RDTs (Determine and Bioline)	26%^c^
Kranzer, 2008	Malawi, rural district	2005–2006	2,047	Factors associated with HBT refusal	11.4%	18–59 y	Trained local VCT counsellors	No	No	Venous blood sampling for ELISA and particle agglutination testing in laboratory	36%
Lugada, 2010	Uganda, five rural districts	2005–2007	4,798	Uptake of HBT versus clinic-based testing in household members of HIV-positive index patient	5.6%	Any	Trained lay field workers	No	No	FP RDTs (Determine screening, Unigold confirmation)	Not specified
Maheswaran, 2012	South Africa, rural district	2009	1,726	Uptake of HBT and community mobile HIV testing and factors associated with HBT versus mobile testing	22%	≥15 y	HIV Counsellors	No	No	Not specified^d^	40%
Matovu, 2002	Uganda, rural district	1999–2000	11,709	Uptake of HBT and effects on sexual risk behaviour and HIV acquisition	*5.6%*	15–49 y	Counsellors	No	No	Venous blood sampling for ELISA (×2) testing in laboratory	55%
Menzies, 2009	Uganda, setting not specified	2003–2005	49,470	Comparison of four testing approaches: door-to-door HBT, household member (of index HIV patient) targeted HBT, stand-alone, hospital-based VCT	*5.6%*	Any	Not specified	Yes	No	FP RDTs (screening test followed by confirmation if HIV-positive; tests not specified)	10%
Michelo, 2006	Zambia, one rural, one urban district	2003	5,445	HIV prevalence survey	*20.4%*	15–59 y	Not specified	No	No	Bionor saliva test and “serum test” for saliva-positive or second saliva test	Not specified
Molesworth, 2010	Malawi, rural district	2007–2008	16,894	To assess the performance of HIV RDTs in a HIV prevalence survey	11.6%	≥15 y	Non-laboratory basic health personnel	Yes	No	Venous blood sampling for RDTs (Determine and Unigold in parallel pre-May 2008, serially post-May 2008)	Not specified
Negin, 2009	Kenya, rural province	2008	2,033	Feasibility, acceptability, and cost of HBT	7.8%	15–49 y	Lay counsellors	Yes	No	FP RDTs (Determine and Bioline)	Not specified
Sekandi^e^, 2011	Uganda, urban district	2009	588	Uptake of HBT and factors associated with HBT	*6.5%*	≥15 y	Trained nurse counsellors	No	No	FP RDTs (Determine screening, Statpak confirmation)	61%
Shisana, 2004	South Africa, nationwide	2002	9,963	HIV prevalence survey	*26.5%*	≥2 y	Nurses	No	Yes—money provided to head of household	FP onto filter paper; ELISA (×2) testing in laboratory	Not specified
Tumwesigye, 2010	Uganda, rural district	2004–2007	282,857	Acceptability and uptake of HBT	*5.4%*	>14 y and eligible children >18 mo^f^	Counsellor and laboratory assistant teams	Yes	Yes—HIV-positive provided with condoms, insecticide-treated bednets, and home water treatment equipment	FP RDTs (Determine screening, Statpak confirmation)	9%
Welz (1), 2007	South Africa, rural district	2003–2004	19,867	HIV prevalence survey (residents)	*27.9%*	Women 15–49 y; men 15–54 y	Trained fieldworkers	No	No	FP onto filter paper; ELISA (×2) testing in laboratory	Not specified
Welz (2), 2007	As above	As above	916	HIV prevalence survey (subset of migrants in the community)	As above	As above	As above	No	No	As above	Not specified
Were, 2003	Uganda, rural district	Not specified	2,373	Uptake of VCT and HBT	*4.1%*	Any	Not specified	No	No	Venous sampling, tests not specified	Not specified
Were, 2006	Uganda, two rural districts	2003–2004	3,338	HIV prevalence and acceptability of HBT among household members of HIV-positive index patient	*4.1%*	Any	Counsellors	No	No	FP onto filter paper; ELISA (×2) testing in laboratory; for children <24 mo, HIV DNA measurement on dried blood spot	4.9%
Wolff^g^, 2005	Uganda, rural (15 villages)	2001	1,591	Uptake of HIV results from HIV prevalence survey	*7.9%*	≥15 y	Counsellors	No	No	Venous blood sampling for ELISA (×2) testing in laboratory	Not specified

a Data from study area, or Joint United Nations Programme on HIV/AIDS national data (adult prevalence) if shown in italics.

b Eligible if <13 y and mother HIV-positive, mother HIV status unknown, or mother dead.

c 35,815/137,268 encountered in the area.

d Stated only as following national guidelines for testing.

e Excluded non-English and non-Lugandan speakers.

f Eligible if mother deceased or HIV-positive.

g Study done in period before antiretrovirals were available.

ELISA, enzyme-linked immunosorbent assay; FP, finger prick; RDT, rapid diagnostic test; VCT, voluntary counselling and testing.

Table 2 summarises the factors that potentially influence the rigour of the studies and shows that there was wide variation in standards of implementation and research. For instance, 11 studies did not describe their sampling strategy, though none showed evidence of selective outcome reporting. Six studies did not automatically provide results to clients upon testing them for HIV (HIV prevalence surveys), and three studies did not report whether return visits were made when individuals were not at home. One study reported giving advice for repeat testing after 3 mo to people testing HIV-negative [26]. Two other studies reported giving HIV prevention counselling to HIV-negative individuals [31],[40]. Ten studies reported some means of linkage to care, mostly advising HIV-positive patients to seek care at the nearest health facility [26],[28],[30]–[32],[36],[39]–[41],[44]. One study presented data on the proportion of individuals linked into care upon testing HIV-positive (*n* = 11,359), with 97% of them initiating co-trimoxazole and 11% commencing antiretroviral therapy [32]. Two studies presented information on the clinical condition of individuals found to be HIV-positive [30],[32]. Following HBT, the majority of HIV-positive individuals who had CD4 counts assessed had measures above treatment initiation thresholds applicable at the time (>200 cells/mm^3^) [30],[32].

**Table 2:** Assessment of study rigour.

First Author, Publication Year	Study Process Quality Indicators									Research Method Quality Indicators	
	Pre-Test Counselling Done^a^	Consent Provided	Test Offered with the Intention of Giving Results to Clients^b^	Confirmatory Laboratory Testing Done	Discordant Results Addressed^a^	Repeat Sampling if Discordant	Repeat Visits if Absenteeism	Specific Advice if HIV Result Negative	Linkage to Care for HIV-Infected	Sampling Strategy Described	Selective Outcome Reporting
Angotti, 2009	Yes	Yes	Yes	No	Not specified	No	No	Yes—retest in 3 mo time	Yes	Yes	No
Choko, 2011	Yes	Yes	No	No	Yes	Yes	No	No	Yes	Yes	No
Helleringer, 2009	Yes	Yes	No	No	Not specified	Yes	Yes	No	No	Yes	No
Kimaiyo, 2010	Yes	Yes	Yes	No	Yes	Yes	Yes	Yes—behaviour change and “ABCs” of HIV prevention	Yes	No	No
Kranzer, 2008	Yes	Yes	Yes	Yes	Yes	Yes	Yes	No	No	Yes	No
Lugada, 2010	Yes	Yes	Yes	No	Yes	Yes	Yes	No	Yes	Yes	No
Maheswaran, 2012	Yes	Yes	Yes	No	Not specified	No	No	No	Yes	No	No
Matovu, 2002	Yes	Yes	No	Yes	Yes	Yes	Yes	No	No	No	No
Menzies, 2009	Yes	Yes	Yes	Yes	Not specified	Yes	No	No	Yes	No	No
Michelo, 2006	Yes	Yes	Yes	Yes	Yes	Yes	No	No	No	Yes	No
Molesworth, 2010	Yes	Yes	Yes	Yes	Yes	Yes	No	No	Yes	Yes	No
Negin, 2009	Yes	Yes	Yes	No	Yes	Yes	No	No	Yes	No	No
Sekandi, 2011	Yes	Yes	Yes	No	Yes	Yes	No	Yes—HIV prevention counselling	Yes	Yes	No
Shisana, 2004	Not specified	Yes	No	Yes	Not specified	Yes	Yes	No	No	Yes	No
Tumwesigye, 2010	Yes	Yes	Yes	Yes	Yes	Yes	No	No	Yes	No	No
Welz, 2007	Not specified	Yes	Yes	Yes	Not specified	Yes	Yes	No	No	Yes	No
Were, 2003	Yes	Yes	No	No	Not specified	No	Yes	No	No	No	No
Were, 2006	Not specified	Yes	Yes	Yes	Yes	Yes	No	No	No	No	No
Wolff, 2005	Not specified	Yes	No	Yes	Yes	Yes	No	No	No	Yes	No

a Where no information is available “not specified” is indicated for these variables, as we considered it possible that these activities were done but not reported in the paper.

b Some studies offered testing but results were not promised, e.g., results available only if client sought the result separately; some studies were entirely blinded, e.g., where testing was done for anonymous population HIV prevalence estimation.

### Proportion of Individuals Accepting Testing and Receiving Results

A total of 524,867 people were offered HBT across the 21 studies, which ranged in size from 216 [36] to 282,857 [32] people. Twelve studies disaggregated data on offer of HBT by gender, with 180,942 men and 198,042 women offered testing overall [27]–[33],[36]–[38],[43]. The proportion of those offered testing who were men (in the studies that reported on gender) ranged from 22% to 49%, with an overall proportion of 47%.

**Figure 2:**
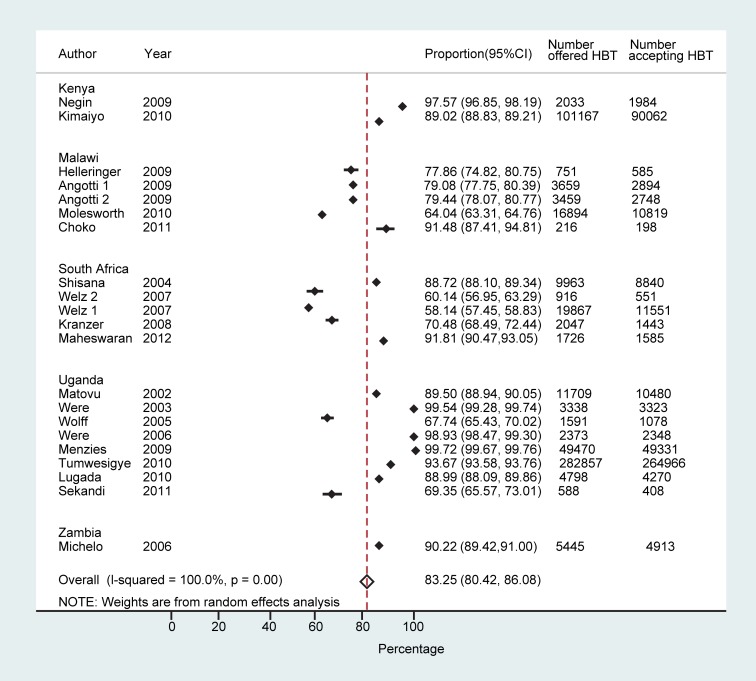
Proportion accepting HBT.

Across all 21 studies the proportion of people who accepted HBT ranged from 58.1% (95% CI: 57.5%–58.8%) to 99.7% (95% CI: 99.7%–99.8%), with a pooled proportion of 83.3% (95% CI: 80.4%–86.1%) accepting to be tested (*n* = 474,377) (Figure 2). Heterogeneity was high (τ^2^ = 0.11). In studies that reported on acceptance of HBT by gender (eight studies) [22],[27],[31]–[33],[38],[43], men were as likely as women to accept testing (78.5% [95% CI: 71.1%–86.0%] versus 81.5% [95% CI: 72.9%–90.1%]). The pooled odds ratio of men accepting HBT was 0.84 (95% CI: 0.56–1.26) compared to women (τ^2^ = 0.33). Studies that offered targeted HBT to household members of index HIV-positive individuals [28],[33] achieved higher proportions of uptake than the other studies: 94.0% (95% CI: 82.4%–100%) versus 80.6% (95% CI: 77.2%–84.0%) (*p*<0.001).

**Figure 3:**
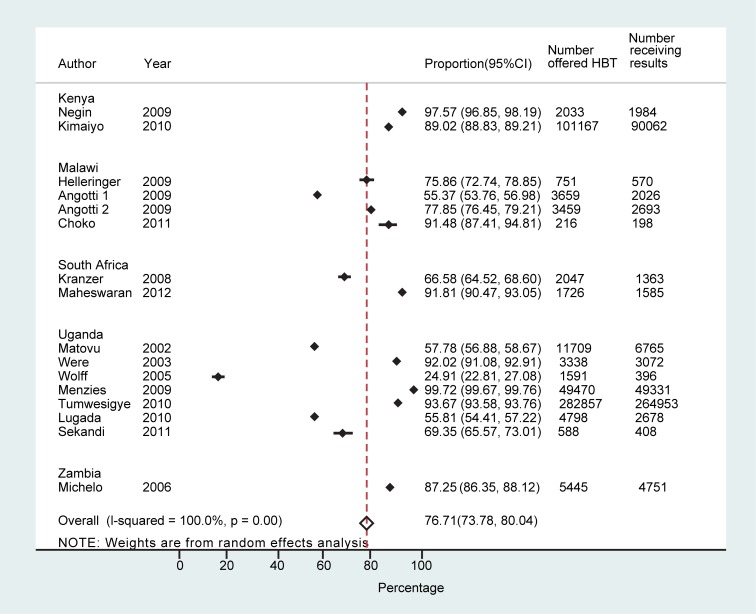
Proportion achieving knowledge of HIV status overall.

Sixteen studies reported on the number of people who received the result of HBT (*n* = 432,835) [26],[28]–[32],[34]–[38],[40],[41],[43],[44]. The proportion receiving a result out of those who accepted testing ranged from 36.8% (95% CI: 33.9%–39.7%) [34] to 100% (95% CI: 100%–100%) [40], with a pooled proportion of 99.6% (95% CI: 99.5%–99.6%) receiving their result (τ^2^ = 0.12) (Figure S1). The proportion of individuals receiving their results overall (out of all those offered testing) ranged from 24.9% (95% CI: 22.8%–27.1%) to 99.7% (95% CI: 99.7%–99.8%), with a pooled proportion of 76.7% (95% CI: 73.4%–80.0%) (τ^2^ = 0.12) (Figure 3).

Eleven studies (*n* = 456,283) reported on the number of individuals offered testing who had already been previously tested (*n* = 78,527) [26],[29]–[33],[36]–[38],[40],[44]; three studies reported on the number tested within the last 12 mo [26],[31],[34]. However, authors did not report the definition of “previously tested” and whether it included all those who had had a test or was limited to those who received their result and became aware of their HIV status. The proportion of individuals previously tested ranged from 5% to 66% overall (11 studies); 22%–50% were previously tested within the last 12 mo (three studies). Studies in which <30% of people had previously been tested (five studies, *n* = 436,618) [30],[32],[33],[37],[40] on average reported a higher frequency of test acceptance than studies in which ≥30% of people had been previously tested (six studies, *n* = 19,665) [26],[29],[31],[36],[38],[44] (92.1% [95% CI: 87.8%–96.4%] versus 82.1% [95% CI: 75.6%–88.6%], *p* = 0.01).

One study explicitly reported excluding individuals already known to be HIV-positive [40]. Angotti et al. [26] reported that 68% (11/72) of known HIV-positive individuals accepted HBT versus 90% (1,430/1,588) of individuals who were HIV-negative when they previously tested. Choko et al. [36] invited participants to partake in oral self-testing even if they knew they were HIV-positive (19 HIV-positive out of 175 previously tested individuals). Amongst individuals previously tested for HIV who accepted HBT in the study by Matovu et al. [22] 10% (*n* = 350/3,362) were already known to be sero-positive. Of those testing HIV-positive through HBT, 40%–79% had not previously been diagnosed (five studies) [22],[31],[33],[36],[40] (this information for the study by Matovu et al. was obtained from a second publication in 2005 [22] rather than the 2002 publication [29] about the same study that was included in this review).

Table S1 summarises the individual-level factors associated with uptake of testing, and shows a wide variation in findings across the studies that reported on this [28],[29],[31],[32],[37],[38].

### Potential Harm and Cost Considerations

Eight of the articles we examined acknowledged the potential for harm from testing for HIV [28],[30],[32],[34],[35],[38],[41],[42], but none reported any harm. Four of these described no adverse events and suggested that HBT could serve to normalise HIV testing by its uniform and non-discriminatory deployment regardless of risk factors or health status [28],[30],[32],[34]. Wolff et al. presented qualitative research findings that fear of stigmatisation and emotional vulnerability associated with receiving results from public facilities were the most common reasons given for the relative popularity of HBT [34]. A further three articles noted that concerns about stigma and fears about confidentiality could account for non-participation in HBT [38],[41],[42]; uptake in these studies was 71%–98%. Another study commented that confidentiality may be enhanced with HBT [35]. Two studies (both from Uganda) reported on the costs of HBT and demonstrated that the cost of testing per client was less than US$9 [30],[32].

### Heterogeneity

**Figure 4:**
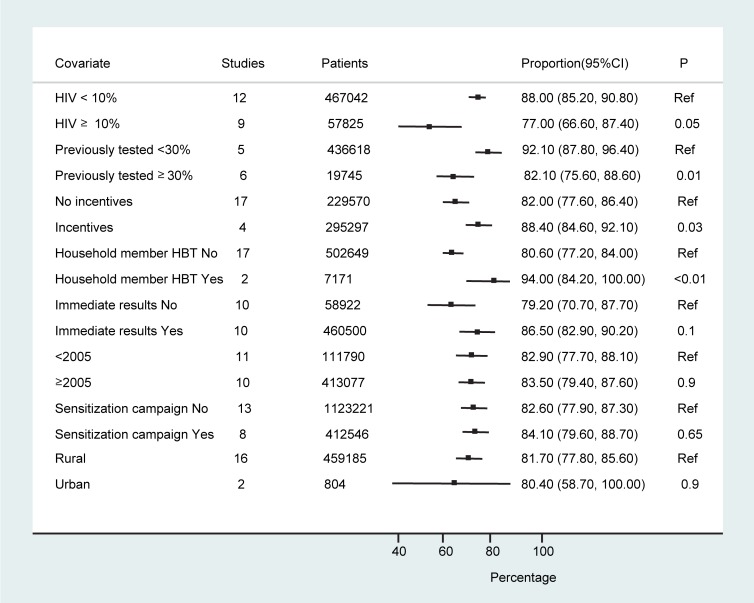
Sub-group analyses.

The studies were conducted in a range of countries, settings, and contexts (of HIV awareness and treatment availability). There were 14 studies examining the feasibility, acceptability, and uptake of HBT as an approach to testing; six studies were carried out to estimate HIV prevalence and utilised HBT as the approach in their surveys; and one study was done to assess the performance of rapid tests for HIV in the context of an HIV prevalence survey. Statistical heterogeneity was also high; however, over three-quarters of the studies (16/21 studies; *n* = 449,970) reported an acceptance rate above 70%. Sub-group analyses to examine heterogeneity did not find any statistically significant differences in HBT uptake and receipt of results according to study period, study setting, or whether sensitisation campaigns were reported as being done (Figure 4). The provision of incentives appeared to result in higher test uptake. Studies in which <30% of individuals had been previously tested, in sites where local HIV prevalence was <10%, or where HBT was offered to household members of HIV-positive individuals also had higher uptake of testing (Figure 4).There was also a tendency towards a greater frequency of test acceptance when immediate provision of results was available, although this finding was not statistically significant (86.5% [95% CI: 82.9%–92.0%] versus 79.2% [95% CI: 70.7%–87.7%], *p* = 0.1).

## Discussion

This systematic review and meta-analysis of 19 papers based on 21 studies of HBT across five countries in SSA demonstrates that voluntary counselling and testing for HIV at home is highly acceptable. While pooled estimates derived from heterogenous studies should be interpreted with caution, an average 83% of people accepted testing, and 99% of those accepting testing received their result. Over three-quarters of everyone who was offered a test accepted to be tested and received their result (77% in 16 studies reporting on this). The proportion of previously undiagnosed HIV was high (40%–79% of those diagnosed HIV-positive), emphasising the value of HBT.

It is acknowledged that means of increasing access to testing are needed in order to achieve universal knowledge of HIV status [5]. A study from Malawi of clinic-based HIV counselling and testing showed that just 13.3% of 18,021 clinic attendances (8.5% amongst men) included HIV counselling and testing [45]. Recent studies have suggested that there is high willingness to participate in HBT, and the proportion of individuals ever tested for HIV in a community in Uganda rose from 19% to 62% following an HBT campaign [6],[46]. This meta-analysis confirms that HBT is an important approach to improve awareness of HIV status in SSA, and it can be used in addition to other approaches such as stand-alone testing, community and work-place testing, and provider-initiated testing.

Delayed presentation for HIV treatment services is recognised as an important cause of morbidity and mortality from HIV despite major progress in increasing access to antiretrovirals [47]. Both studies that reported on the clinical status of patients diagnosed HIV-positive upon HBT found that the majority had CD4 counts above treatment initiation thresholds (for the study period) [30],[32]. Tumwesigye et al. found that of the HIV-positive individuals tested for CD4 count, 45% had CD4 count >350 cells/mm^3^ (and 68% >200 cells/mm^3^) [32]. Similarly, Menzies et al. found that 69% of HIV-positive individuals identified through HBT had CD4 count >200 cells/mm^3^. In this latter study, which compared approaches of HIV testing, the proportions of HIV-positive individuals identified with a CD4 count <50 cells/mm^3^ through stand-alone voluntary counselling and testing and hospital-based testing were 20% and 24%, respectively, while the corresponding proportion was 12% for HBT targeted to household members of known HIV-positive individuals, and 6% for untargeted HBT [30]. This is consistent with other findings that suggest HBT is a useful approach for earlier detection of HIV, initiation of treatment, and better prognosis [13],[48], as well as for higher impact with treatment as prevention [49],[50]. A recent pilot study in South Africa found a reduction in mean community viral load 6 mo after the introduction of a HBT campaign [51].

While women are disproportionately affected by HIV in SSA [5], men have long been known to under-utilise HIV services and to present later for care than women, and consequently they have worse outcomes on treatment [45],[52],[53]. In the studies reviewed here, an overall proportion of 47% of those offered testing were men. This compares favourably with facility-based testing, where testing of males attending the clinic may be as low as 9% [45]. In our meta-analysis of HBT, an almost equivalent proportion of men were offered a test as women, and they were as likely to accept testing, an outcome that gives promise of greatly improving awareness of HIV status for both sexes. Studies that provided results at a distant site even if testing was conducted at home were associated with lower proportions of people receiving results out of those who accepted testing. While this emphasises the benefits of HBT including immediate provision of results in raising awareness of HIV status, it may be of less concern given that rapid diagnostic tests with immediate results are now the norm for voluntary HIV testing globally.

Examination of trends by country suggest lower uptake of testing in South Africa, where three out of five studies reported uptake of ≤70% (note that two of these studies were in the same setting in KwaZulu-Natal) (Figure 1) [27],[38]. However, the most recent study from South Africa found very high uptake of HBT (91.8%) [44]. Based on the paucity of countries and the number of studies per country available for this review, it would be unwise to draw conclusions about country differences and acceptability of HBT.

While the results of sub-group analyses need to be interpreted with caution, they suggest that the running of pre-test sensitisation campaigns may be of little benefit in terms of uptake of HBT. However, these are essentially “ecological” comparisons, which may be confounded by many other differences between the study populations examined. Also, the number of studies where incentives were given was very small (Table 1), and strong conclusions cannot be drawn. Nevertheless, the fact that most of the studies demonstrated similar proportions of uptake of HBT perhaps argues against a strong effect. The finding that studies with a lower proportion of individuals previously tested for HIV (<30%) had a higher frequency of test uptake points to the value of HBT as an effective approach to engage those not previously aware of their HIV status in testing. It could suggest that HBT is effective in achieving initial diagnosis but less so for repeat testing. Targeted HBT of index HIV-positive clients' household members may be an effective way to achieve higher acceptance in settings where more general HBT is not feasible because of resource limitations.

Uptake of HBT may be influenced by availability of treatment, as indicated by the fact that the study with the lowest overall success (only 25% of people offered a test received their result) was done at a time when antiretroviral treatment was not available in the communities studied [34] (although overall there was no effect of “study period”). However, there may be other confounding factors involved, and this study was based on a small sample size; in sensitivity analysis, excluding it from the analysis did not change the pooled estimate of uptake of HBT (data not shown). Three other studies were notable for having <70% receipt of results amongst those who accepted HBT (Figure S1). Two of these studies offered the option of receiving results at a later date [26],[29], while the third [28] offered the option of receiving results on the same day.

Human rights protections should be an integral part of any testing campaign, and every effort should be made to avoid physical, social, and psychological harm to individuals [16],[17]. However, the high level of uptake we have found overall seems to indicate acceptability of HBT in the communities studied.

There are several strengths and limitations to this review. We used a broad search strategy that allowed us to capture 21 studies (published in 19 articles), resulting in a large overall sample size and giving increased confidence in the pooled estimates. There was high statistical heterogeneity, as expected for pooled proportions in observational studies. We limited our search to studies conducted in SSA over the last decade in order to improve comparability, and used a random-effects model to pool data. We undertook a number of sensitivity and sub-group analyses to explore potential sources of heterogeneity. The non-uniformity of the studies, which were nonetheless looking at uptake of a “uniform” activity (the offer of an HIV test at home), could be considered both a strength and limitation of our review. While it may be a limitation for pooling results, it could be considered a strength that even in a range of study contexts, HBT consistently achieved higher uptake than is seen in facility-based testing.

Another limitation was that, as a trade-off to using a broad search strategy, our search was limited to just three databases and to published articles in peer-reviewed journals. We therefore cannot rule out the possibility that we may have missed some studies, or the possibility of publication bias leading to the non-publication of studies with lower uptake. The limited number of studies that provided data on the health status of those identified as HIV-positive by HBT is a further shortcoming that this review was unable to address. Our findings do, however, indicate a number of directions for future research. In particular, key areas for research include linkage to care following HBT, retention in care of those identified HIV-positive through HBT (who are more likely to be clinically well when diagnosed), as well as repeated HBT for ongoing knowledge of HIV status. The option of self-testing with support from HBT staff is an area of research that is highly topical given recent developments in self-testing [54]. The suggestion from this review that the conduct of sensitisation campaigns has little or no impact on uptake of HBT and receipt of results has important implications for programme cost and efficiency and deserves further evaluation. More data are needed on the effectiveness of HBT in detecting previously undiagnosed HIV infection. Sustainability and cost considerations (short- and long-term) are important to help guide policy, and further work on cost-effectiveness is required. Further research on individual-level factors associated with participation in HBT, such as that recently published by Cherutich et al. [6], would inform implementers on individuals who require further engagement to encourage uptake.

A key finding of our review is that HBT is able to reach wide sections of communities in a diverse range of contexts and settings. HBT provides the opportunity to acquire knowledge of HIV status at the doorstep for those who may not otherwise have sought testing, and may be pivotal in providing an effective tool for governments and health service providers to increase access to HIV treatment and prevention, by increasing uptake of testing. We conclude that HBT has the potential to dramatically increase awareness of HIV status in previously undiagnosed men and women in SSA. HBT is a gateway to accessing care early, and the benefits for individual and public health, both for treatment and prevention, make it an invaluable tool in the fight against HIV.

## Supporting Information

Figure S1 **Proportion receiving result of HBT.**
(TIFF)

Table S1 **Studies reporting individual-level predictors of uptake of HBT.**
(DOCX)

Text S1 **PRISMA statement.**
(DOCX)

Text S2 **Search protocol.**
(DOCX)

## Abbreviations

HBT: home-based voluntary counselling and testing
SSA: sub-Saharan Africa
