# Supplementary figures and images for: Uptake of Home-Based Voluntary HIV Testing in Sub-Saharan Africa: A Systematic Review and Meta-Analysis
Source: PLoS Med. 2012 Dec 4;9(12):e1001351. doi: 10.1371/journal.pmed.1001351 (PMC3514284; doi:10.1371/journal.pmed.1001351)

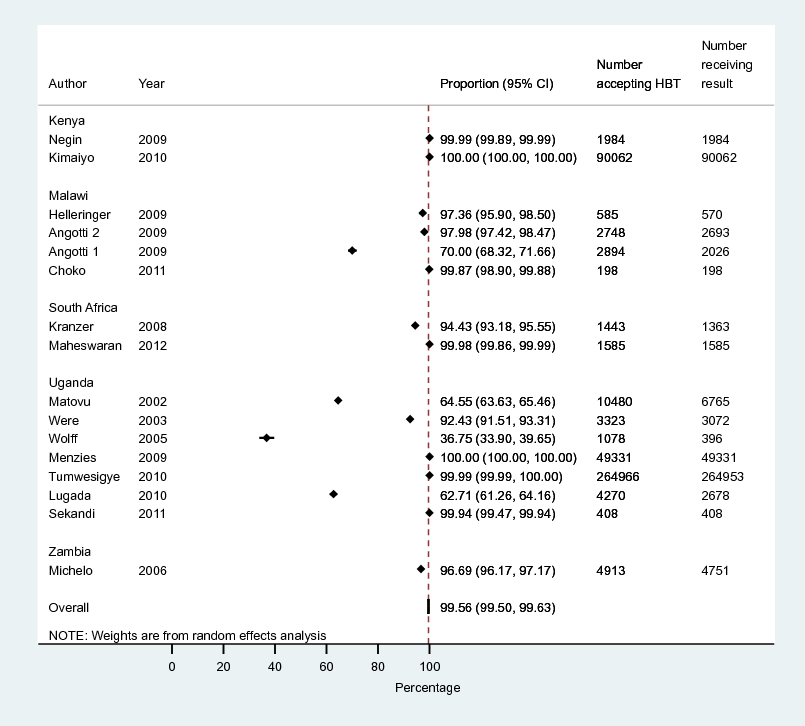

Supplement: Figure S1 — Proportion receiving result of HBT. (TIFF) [file pmed.1001351.s001.tif]
