# Supplementary material for: Uptake of Home-Based Voluntary HIV Testing in Sub-Saharan Africa: A Systematic Review and Meta-Analysis
Source: PLoS Med. 2012 Dec 4;9(12):e1001351. doi: 10.1371/journal.pmed.1001351 (PMC3514284; doi:10.1371/journal.pmed.1001351)
Supplement: Table S1 — Studies reporting individual-level predictors of uptake of HBT. (DOCX) [file pmed.1001351.s002.docx]

| **Author, Publication year** | **Positive association with HBT uptake** | **Negative association with HBT uptake** | **No statistically significant association (aOR)** | **Comments** |
| --- | --- | --- | --- | --- |
| Helleringer,  2009 | Income bottom quartile;  Symptomatic STI in last 3m | Age>25y;  Having concurrent partnership at time of HBT | Gender;  Marital status;  Schooling;  Religion;  Residence mainland;  No. of sexual pertners past 3y;  Ever tested prior to study |  |
| Kranzer,  2008^a^ | Female (Odds Ratio not given);  Never married;  Farmer profession;  Older (>45y) head of HH^b^;  >7% HIV prevalence in cluster | Female counsellors approaching male clients;  Wife of head of HH^b^ man who's not tested OR Wife of head of HH^b^ man who's not part of study OR head of HH^b^ is non-husband;  <1km from main road | Age | Men less likely to be found at home |
| Lugada,  2010 | Female;  Age: <14 or >35y when compared with 15-24 | Index HIV-positive client CD4 >200 (compared to <50) | Index client education level;  No. of persons in HH ^b^ |  |
| Matovu,  2002 | Currently married or Divorced/widowed/seperated compared to never married | Primary/post primary education compared to no education;  Prior self reported VCT or no prior VCT compared to prior VCT in the programme;  HIV +ve vs HIV-ve;  Condom use vs no condom use in past 6m | Age;  Gender;  Self perception of HIV risk;  No. of sexual partners |  |
| Sekandi, 2011 | Male;  Age ≥35y compared to 15-24y;  Previously married;  Previous HIV testing in last 12m | Not Applicable | Age 25-34y:  Religion;  Education level;  Previous HIV testing > 12m prior |  |
| Tumwesigye, 2010 | Female | Not Applicable | Not Applicable | Females more likely to be found at home |

^a^ Study actually reports refusal of HBT ^b^ Household
