# Supplementary material for: Uptake of Home-Based Voluntary HIV Testing in Sub-Saharan Africa: A Systematic Review and Meta-Analysis
Source: PLoS Med. 2012 Dec 4;9(12):e1001351. doi: 10.1371/journal.pmed.1001351 (PMC3514284; doi:10.1371/journal.pmed.1001351)
Supplement: Text S2 — Search protocol. (DOCX) [file pmed.1001351.s004.docx]

**Search protocol for**

**Home-based voluntary HIV testing in sub-Saharan Africa: a systematic review and meta-analysis**

**Background:**

Testing for HIV is the first step in the cascade of care for HIV-positive individuals who need treatment. Knowledge of HIV status is also an important part of HIV prevention, for both HIV-negative and HIV-positive individuals, and innovative means to increase uptake of testing has recently been identified as an international policy priority. Despite some progress, knowledge of HIV status remains low in sub-Saharan Africa (SSA) where HIV prevalence is highest. Men are less aware of their status than women (in countries with available data).

Home-based voluntary counselling and testing (HBT) has recently been suggested as an effective way to identify HIV-infected people earlier in the stage of their disease and so enrol people into care and treatment in a timely manner.

This study will be a systematic review and meta-analysis of the available evidence for acceptability of HBT in SSA. It will assess a number of potential determinants of uptake and programme success.

**Search terms:**

1. HIV
2. Voluntary counselling and testing
3. Home based
4. Mobile
5. Community
6. Workplace
7. Couples
8. Self
9. Africa
10. (1 and 2)
11. 10 and (3 or 4 or 5 or 6 or 7 or 8)
12. 11 and 9

**Databases:**

1. Pubmed
2. Embase
3. Global Health

**Restrictions:**

No language restrictions will be applied

**Inclusion criteria:**

1. Age range: any
2. Dates: 1^st^ January 2000 - 24^th^ September 2012 (time of most up to date search)
3. Language: any
4. Geographic range: SSA
5. Study design: RCTs, observational cohort studies, cross-sectional surveys, programme evaluations
6. Setting: service delivery in any SSA country through research intervention, MoH, NGO or other private initiative
7. Minimum information included: report on home-based offer of HIV test approach deployed and proportion accepting testing

**Exclusion criteria:**

1. Dates: before January 2000
2. Topic: not on HIV testing approaches in SSA
3. Study setting: exclude if testing in any HC facility (hospital/health centre/mobile clinic) OR VCT centre
4. Study design/analysis: modelling studies; qualitative studies, surveys examining predictors of having a test in general (willingness/knowledge/acceptability etc)
5. Exclude if minimum information NOT included: ie proportion accepted test

**Objectives:**

The specific objectives of the study will be to summarise the following proportions: “accepted” (or uptake), defined as accepted and had HIV test performed at home as a proportion of all individuals offered HBT; “received”, defined as obtained result of home-based HIV test as a proportion of all individuals who accepted; and “overall” defined as the proportion of patients who received a test result among all those offered VCT (including refusals).

**Study process quality indicators:**

1. Consent provided
2. Test offered based on giving results (rather than offer of testing without promise of results)
3. Confirmatory laboratory testing done
4. Repeat sampling if discordant
5. Repeat visits if absenteeism
6. Discordant results addressed
7. Specific advice if HIV result negative
8. Linkage to care for HIV infected

**Research method quality indicators:**

1. Sampling strategy described
2. Selective outcome reporting present

**Data analysis**

We will calculate point estimates and 95% confidence intervals of the proportion of people who accepted HIV testing at home and the proportion who received their test result, out of those i) who were offered and ii) who accepted testing. The variance of raw proportions will be stabilised using a Freeman-Tukey type arcsine square-root transformation and proportions were then pooled using a DerSimonian-Laird random-effects model. Pooled odds ratios will be calculated for proportions stratified by gender using the same method.

**Heterogeneity**

We will report the *I^2^* statistic to assess the proportion of variability due to between-study heterogeneity (but this estimate is known to increase as the number of subjects contributing to the meta-analysis increases[^13^](#_ENREF_13)). We will therefore also report τ^2^ as a measure of between-study variance (reporting on the arcsine square-root scale). We will explore potential sources of heterogeneity through univariate subgroup analysis to determine the potential influence of the following covariates: HIV prevalence (<10% vs ≥10%), study period (<2005 vs ≥2005), incentives provided, sensitization campaigns done, and study setting (urban vs rural), potential influence of type of test (point-of-care testing with immediate result and whether oral specimens were used), Finally, further subgroup analyses will be done to assess the potential influence of the proportion of individuals in the study who had previously tested (to be arbitrarily divided into 2 categories, <30% vs ≥30%), and targeted HBT of household members of index HIV-positive individuals.

**Statistical software**

All analyses will be conducted using Stata version 12.0 (Stata Corp, College station, Texas).
